# Evaluation of serum proteome from Indian psoriasis patients

**DOI:** 10.6026/97320630018141

**Published:** 2022-03-31

**Authors:** Saiqa R Shah, Varshasnata Mohanty, Debajit Chaudhury, T.S. Keshava Prasad, Manjunath Mala Shenoy

**Affiliations:** 1 Yenepoya Medical College, Yenepoya (Deemed to be university), Deralakatte, Mangalore, India; 2 Institute of Life sciences, Bhubaneswar, Odisha, India; 3 Stem Cell and Regenerative Medicine Centre, Yenepoya Research Centre, Yenepoya (Deemed to be University) India; 4 Centre for Systems Biology and Molecular Medicine Yenepoya Research Center, Yenepoya (Deemed to be University) India

**Keywords:** Psoriasis, LC-MS/MS, Orbitrap, biomarkers, serum

## Abstract

Psoriasis is a polygenic chronic skin condition, associated with many systemic disorders. Though it is most studied dermatological condition, molecular mechanism leading to its pathogenesis is still unclear. An insight into its proteome may help unrevealing some biomarkers and therapeutic targets. In this study, we carried out mass spectrometry based quantitative proteomic analysis of serum from psoriasis patients by employing Tandem Mass Tags (TMT) approach. We identified 861,887 MS/MS spectra corresponding to 493 proteins. These dysregulated proteins were further classified by Gene Ontology and protein-protein interaction of dys-regulated proteins revealed networks in psoriasis patients.

## Background:

Psoriasis is an inflammatory, chronic skin disorder involving immune system. The red, scaly plaques can involve any part of the body, but extensor surfaces are most involved. The characteristic histo-pathological features include parakeratosis, hyperkeratosis with immune cell infiltration [[1]. There is an atypical immune response in genetically predisposed persons involving 2% of world's population [2]. Several factors like alcohol consumption, smoking, injury, stress, diet and drugs have been observed as a trigger to flare up the disease [3].There are different clinical types, plaque, erythrodermic, pustular, and inverse psoriasis with 90% of patients having plaque type [2]. Age distribution varies, however, two peaks with bigger one at 20-30 years and a smaller peak at 50-60 years is seen [4]. Psoriasis is not restricted to skin, but is linked to systemic disorders like metabolic syndrome, obesity, dyslipidemia, etc which worsen its prognosis [5]. Various epidemiological studies have shown that psoriasis patients have a higher mortality rate when compared to healthy individuals and life expectancy decreases by 5 years in patients with increase in severity. The cardiovascular co morbidities increase the burden on economy as well as on the healthcare [6]. Psoriasis management requires cautionary skin care as the co morbidities are observed in patients with type 2 diabetes, metabolic syndrome, obesity, poor quality of life, and depression [7]. T-helper 1 (Th1), T-helper 17 (Th17) cells and their related cytokines are implicated in pathogenesis of psoriasis but a complete understanding is lacking which promote lesion development. Evidences support the abnormal activation of Toll-like receptors (TLRs) which lead to initiation of psoriasis and hence targeting TLR7, 8, and 9 will help in neutralizing the multiple inflammatory pathways and target the individual cytokines for treating the autoimmune diseases like psoriasis [7]. Previous studies have focused on inhibiting activation of (mTOR)/S6K pathway which have only limited efficacy for treatment of psoriasis but recent findings on the mechanism underlying hyper-translation have highlighted involvement of proteins besides canonical mTOR targets [8]. Therefore by targeting definite stages of translation initiation, elongation or termination, novel therapeutic responses may be obtained. In case of psoriatic plaque, the cytokines are represented by high serum levels of TNF-α downstream cytokines, IL-6 and IL-8, are spread into the circulation. In non-lesional skin, the inflammatory cytokines may provoke downstream genes in IL-17 signalling pathways to produce a disease phenotype. The available biologics for psoriasis basically target the function of TNF-α, IL-17A or IL-12/23 includes etanercept (anti-TNF receptor fusion protein), adalimumab and infliximab (anti-TNFα antibodies), anti-IL-17(receptor) molecules and ustekinumab [9]. The occurrence and severity of psoriasis has also reported in patients with obesity where an increased level of Osteopontin (OPN), TNF, CCL5 and CXCL9 levels are documented [10]. Recent studies have showed elevated level of Interleukin-6, 8, 17A, 22, 23, and TNFα in psoriasis patients as compared with controls. In the sensitivity studies, patients with active psoriasis showed extensively increased levels of IL-17A, IL-23, and IL-22 as compared to the group of patients with stable psoriasis [11]. The psoriasis lesional skin transcriptome studies have revealed a better understanding of activated cellular pathways within lesions which can help in designing new disease mechanism and possible drug targets [8] but changes in mRNA expression alone cannot reflect protein abundance [12-15]. In order to completely understand the physiological and pathological conditions in psoriasis, trancriptome -proteome integration of differentially expressed mRNA impacting protein abundance approach would be more substantial for the disease process. Thus, this study demonstrated proteomics and bioinformatics strategy which will bring key aspects of psoriasis into focus at the cellular level. This approach will help in unfolding disease-specific and non specific signals further throwing some new insights into pathogenesis of the disease. In this study, we carried out quantitative proteomic analysis using Thermo Scientific Orbitrap Fusion Tribrid mass spectrometer to study the differential protein expression in serum of psoriasis patients as compared to healthy individuals with no symptoms. Our study resulted in the identification of 493 proteins of which 8 proteins were significantly (p≤0.05) up regulated while 12 proteins were significantly (p≤0.05) down regulated.

## Materials and Methods:

### Sample collection:

Whole blood samples were collected from patients diagnosed with psoriasis after obtaining approval from the Institutional Ethics Committee at Yenepoya University, Mangalore, India. For control cases, blood samples were collected from voluntary donors. Prior to collection, written informed consent were obtained from both healthy volunteers and patients with psoriasis. Serum was separated using standard centrifugation techniques and samples were stored at -80°C until further use.

### Protein extraction:

Depletion of abundant proteins in serum samples were carried out using a Human-14 Multiple Affinity Removal Column (Agilent Technologies) as per manufacturer's protocol. Post depletion, protein concentration was estimated using Bicinchoninic acid (BCA) assay (Thermo Scientific Pierce, Rockford, IL, USA). An equivalent amount of protein from each of patients and control serum samples were taken for further analysis.

### Sample preparation for LC-MS analysis:

An equal amount of proteins from each condition was subjected to reduction, alkylation and trypsin digestion. Reduction and alkylation of the proteins was done by using 5mM of dithiothreitol and 20 mM of iodoacetamide, respectively. Trypsin was used as a proteolytic enzyme, in a concentration of 1:20 (enzyme: protein) and digestion was confirmed on SDS-PAGE. Digested peptides were subjected labeling using TMT tags. The labelled peptides were then subjected to fractionation and LC-MS/MS analysis. Data was acquired in technical triplicates.

### Mass spectrometry data analysis:

LC-MS/MS analysis of the samples was carried out using Thermo Scientific Orbitrap Fusion Tribrid mass spectrometer (Thermo Fisher Scientific, Bremen, Germany) using Orbitrap as mass analyser. Acquired raw data were processed on Proteome Discoverer (2.1) Suite with SEQUEST and Mascot as search algorithms against RefSeq human protein database. Search parameters included oxidation of methionine and acetylation of protein N-terminus as variable modification, whereas, carbamido methyl of cysteine as dynamic modification. False discovery rate was calculated using percolator with a reverse database. Mass error window of 10 ppm and 0.05 Da was allowed for MS and MS/MS, respectively. The peptide and protein data were extracted using high peptide confidence (1% FDR). Quantitation was carried out using the reporter ion quantifier node for TMT.

### Bioinformatics analysis:

Differential expression ratios were calculated where a fold change of ≥ 1.35 was considered to be upregulated and fold change of ≤ 0.74 was considered to be down regulated. Data was analyzed using unpaired t-test and p-value <0.05 was considered to be statistically significant. Gene Ontology (GO) analyses for both up- and down-regulated proteins were carried out using PANTHER (www. http://www.pantherdb.org/). Protein-protein interaction (PPI) network of dysregulated proteins were done using STRING software (www.string-db.org).

## Results and Discussion:

In post genomics era, proteomics has become an important tool to study and identify proteins using mass spectrometry [16]. Further bioinformatics analysis is beneficial for analysing the proteins identified in a broader extent [17]. For such investigations, depletion of major abundant proteins such as albumin, keratin is favourable prior to proteomics analysis. Proteomics data leads to a better knowledge of disease biology, physiology, and pathogenic pathways and can further aid in biomarker discovery and identification of novel targets [18,19].

## Identification of differentially expressed proteins:

In the current study, the TMT-labelled samples were analysed on high-resolution mass spectrometry to identify the differentially expressed proteins in psoriasis and control serum samples (Figure 1 - see PDF). The search resulted on the identification of 861,887 MS/MS spectra corresponding to 493 proteins. Based on the fold change cut off (±1.35), we found 12 proteins to be significantly (p≤0.05) down regulated and 8 to be upregulated. To further explore the biological functions of the dysregulated protein, we carried out GO analysis. The upregulated serum proteins were mainly enriched in the cellular process, metabolic process, and biological regulation (Table 1 - see PDF), while the functional enrichment terms of significantly down regulated proteins were mainly correlated with binding activity and catalytic activity (Table 2 - see PDF).

Protein-protein interaction (PPI) analysis of these dysregulated proteins were carried out using STRING software (Figure 2 - see PDF). The analysis was carried out for both up- and down-regulated proteins. For this analysis, we carried out PPI analysis using up- and down-regulated proteins to generate the PPI network. The network has been provided in Figure 2A-B(see PDF). The interaction was derived at high level of confidence (≥ 0.7). We further narrowed the analysis using significantly altered proteins. The analysis of the upregulated proteins revealed that there were 8 nodes and 2 edges based on a high confidence score ≥ 0.7 in STRING analysis (Figure 2a - see PDF). Similarly, for the down regulated proteins, the analysis revealed that there were 12 nodes with 2 edges. The network generated from the significantly upregulated proteins revealed high level of connectivity among 3 proteins such as Lipoproteins a (LPA), C-reactive protein (CRP) and lipopolysaccharide-binding protein (LBP) (Figure 2A - see PDF). We identified significant over expression of LPA (Lipoprotein (a)) in the serum of psoriasis patients as compared to the serum from healthy individuals (Fold change +2.16, p=0.01). Studies have demonstrated association of increased level of LPA with disease severity in psoriasis patients resulting in complications [20]. Higher levels of LPA in serum indicate that psoriasis is linked with oxidative stress, further resulting in disease severity. Increased LPA levels may potentially impact the expression of vascular adhesion protein 1 (VAP-1) or its function in T cell adhesion and migration, making psoriasis and associated consequences more likely [21]. Similarly we also identified increase expression of C-reactive protein (CRP) which is a well known inflammatory marker. Inflammatory nature of psoriasis makes higher expression of CRP more evident in serum of psoriatic patients. Studies have also indicated increased expression of CRP in psoriasis patients, suggesting its crucial role as a diagnostic marker for monitoring disease severity and activity [22-24]. Our study also revealed higher expression of peroxiredoxin 2 (PRDX2) which is involved in Redox balance system in proliferating cells. Similar expression has been reported in various studies [25]. Lipo polysaccharide-binding protein (LBP) serves as reliable marker for serum lipopolysaccharide concentration where a higher level of LPS is known to elicit chronic inflammatory cytokines level. Studies have demonstrated its raised level to be associated with onset of psoriasis [26]. PRDX2 is also known to be a negative regulator of apoptosis and is a defence response-related protein. Despite its active role in psoriasis, the underlying molecular mechanism is yet to be deciphered and hence further studies are warranted. Similarly, the network generated from the significantly down regulated proteins revealed high level of connectivity among 3 proteins such as thymosin beta-4 (TMSB4X), actin (ACTB) and profilin (PFN1) (Figure 2B - see PDF).

We observed significant down regulation of profilin-1 (PFN1), platelet basic protein (PPBP) and selenoprotein P (SELENOP). PFN1 plays a major role in cellular proliferation, motility, and cellular growth and thus any aberrant expression resulting in inflammatory diseases [27,28]. Lower levels of TMSB4X have been identified in the serum of psoriasis patients which also has been reported previously [29,30]. This protein has been known to play an active role in organization of the cytoskeleton and known to bind to and sequesters actin monomers, thus, inhibiting actin polymerization. Past research had shown that both the basement membrane molecular composition and the polarised expression of integrins were altered in psoriatic lesions. In the current study, we observed similar observation where proteins such as ACTB and TMSB4X were observed to be altered in the serum of psoriasis patients as compared to healthy volunteers.

## Conclusion:

Nonetheless, proteomics research in psoriatic disease is still in its early stages as there are still numerous gaps that need to be filled. Validation of putative protein biomarkers in a large cohort of patients is required in order to properly establish diagnostic and prognostic biomarkers that may be employed in clinical practise. Furthermore, proteomics in combination with other emerging approaches will enhance our knowledge of disease mechanisms and, in turn, will aid in identification of therapeutic targets for psoriasis therapy in the future.
